# Lipoprotein(a) and Effects of Diet: Time for Reassessment

**DOI:** 10.3390/nu17101714

**Published:** 2025-05-19

**Authors:** Byambaa Enkhmaa, Lars Berglund

**Affiliations:** Department of Internal Medicine, School of Medicine, University of California Davis, One Shields Avenue, Davis, CA 95616, USA; lberglund@ucdavis.edu

**Keywords:** Lp(a), LDL-C, metabolic effect, saturated fat, simple sugar, ketogenic diet

## Abstract

Dietary modification is a critical tool in the prevention of cardiovascular disease (CVD). While the role of saturated fat (SFA) intake is well established in affecting LDL cholesterol concentrations, diet impacts on lipoprotein(a) (Lp(a)) have been less studied. Lp(a) is a prevalent, strong, and highly heritable risk factor for CVD and a therapeutic target for CVD risk management. While significant insights have been made into the genetic regulation of Lp(a), our understanding of any metabolic impact on Lp(a) by other factors, including diets, is limited. For many years, Lp(a) was not considered to be subject to dietary regulation, but there is now clear evidence of a dietary impact, in particular variability in SFA intake, on Lp(a) concentrations. The present narrative review aims to provide an updated view on dietary regulation of Lp(a), moving beyond studies testing the effect of reducing SFA intake, to include new evidence from clinical trials on the impact of an increased sugar intake and ketogenic diets. In addition to describing an opposite effect of SFA on Lp(a) and LDL cholesterol concentrations, with a rise in Lp(a) during a reduced SFA intake, this review also provides new data on the role of apolipoprotein(a) size polymorphism, a major genetic regulator of Lp(a) concentrations. Beyond an impact on Lp(a) concentrations, the extent to which diet might impact Lp(a)’s molecular and metabolic properties including its lipidomic composition remains unknown. Taken together, evidence shows the presence of a dietary modulation of Lp(a) beyond its genetic control and points to the need to better understand Lp(a)’s cardiovascular risk factor properties, including metabolomics/lipidomics characteristics. This also raises the issue whether diet should be a component of elevated Lp(a) management, and this needs to be addressed in future studies.

## 1. Introduction

Dietary modification has been an important tool for strategies to prevent cardiovascular-related morbidity and mortality. Early trailblazing studies such as the Seven Countries Study contributed to establishing a relationship between dietary saturated fatty acid (SFA) intake and cardiovascular disease (CVD) [1]. This concept has held up over a long observation time as recently demonstrated in a study of 221,054 adults from three large cohorts [2]. In this cohort study, higher intake of butter was associated with increased mortality, while higher plant-based oils intake was associated with lower mortality. Thus, substituting butter with plant-based oils may confer substantial benefits for preventing premature deaths, including CVD mortality [2]. Although reduction of SFA intake has been robustly associated with lower LDL cholesterol (LDL-C) concentrations and a decreased CVD risk, there is more uncertainty regarding optimal replacement strategies [3]. As public health concerns regarding obesity and risk for type 2 diabetes mellitus have increasingly been brought into focus, the previous preference regarding carbohydrates as a replacement choice has been more debated. Although lipoprotein(a) (Lp(a)) has a similar lipid moiety to LDL, it was long considered that diet has only a negligible effect on Lp(a) concentrations. However, given the consistent lowering effect of LDL-C by reducing SFA intake, recent observations that this same intervention strategy results in an increase in Lp(a) concentrations are noteworthy [4].

Lp(a) has emerged as one of the strongest genetically determined risk factors for CVD [5,6,7,8]. Elevated Lp(a) concentrations affect one in five people (15−30% of the population depending on geographical location) [9], making Lp(a) a highly prevalent CVD risk factor. The evolving landscape of Lp(a) research and accumulating clinical evidence for the risk factor role of Lp(a) have shaped recommendations by clinical guidelines to address the Lp(a)-attributable CVD risk [5,6,10] as well as future research directions to fill in the existing gaps in our knowledge base [11]. Notably, recent innovations in Lp(a)-specific drug development have enabled the testing of the Lp(a) hypothesis, i.e., whether intensive lowering of elevated Lp(a) would lead to reductions in cardiovascular outcomes. To date, several drugs with potent Lp(a)-lowering effects (up to 98% maximum reduction in Lp(a)) are in various stages of their clinical developments [12,13,14,15,16,17,18]. While these therapies, if proven successful, will be of significant importance for treatment of very high Lp(a) levels, the questions regarding prevention strategies for a larger population segment remain. Management of elevated Lp(a) needs to be seen as part of a comprehensive CVD risk reduction strategy. As diet remains a foundational tool in this arsenal, it will be increasingly important to assess any role of diet modification for Lp(a) concentrations and atherogenic properties.

Based on the growing evidence from large studies including those in the UK Biobank and Denmark, the Lp(a)-attributable risk is the strongest for myocardial infarction (MI) and aortic valve stenosis (AVS), followed by peripheral artery disease (PAD) and heart failure (HF) [17]. For these conditions, the relative risks are reported to be as high as 1.7 to 3 times for individuals with the top 5% of Lp(a) concentrations. On the other hand, the weakest associations were seen for ischemic stroke and CVD and all-cause mortality, with relative risks of 1.2 to 1.6 times for people with the top 5–10% of Lp(a) concentrations [17]. In contrast, genetically determined low Lp(a) concentrations have been associated with a decreased risk of coronary heart disease (CHD), PAD, stroke, HF, and AVS [19]; at the same time it appears that genetically determined low Lp(a) concentrations do not cause any significant increase in the risks of other disorders, including diabetes and cancer [19]. As the attention to Lp(a) concentrations in CVD risk assessment is increasing, the need for a well-standardized assay methodology for Lp(a) outcomes is becoming even more evident. This is related to Lp(a)’s unique structural variability, i.e., a size polymorphism in its apolipoprotein(a) (apo(a)) component, i.e., a hypervariable number of its Kringle (K) 4 type 2 repeats. The latter is considered as the major inverse genetic determinant of Lp(a) concentrations. Of note, the apo(a) size variability introduces a challenge for accurately measuring Lp(a) concentrations and the necessity of using apo(a)-size-insensitive assays as well as outcomes being measured in particle concentrations (e.g., nmol/L). As the detailed discussion of this methodological issue is beyond the scope of this review, please review details elsewhere [20].

Today, Lp(a) is regarded as a risk trait with a highly complex genetic regulation, where a large number of Lp(a)-lowering and Lp(a)-increasing genetic variants in the *LPA* gene interact to determine the final circulating concentration of Lp(a) [21]. Notably, while there have been many advances regarding genetics of Lp(a), there is less insight into the phenotypic qualities that underlie its atherogenicity. Recent advancements in state-of-the-art technologies offer unique tools to conduct such analyses beyond measurement of its plasma concentration. New metabolomic, proteomic, and lipidomic tools hold promise to critically improve our understanding of the fundamental quantitative and qualitative risk properties of Lp(a) as well as testing of their responses to pharmacological and lifestyle interventions, including diet.

## 2. Lp(a) and Lowering of Dietary Saturated Fat Intake

Given the public health importance of diet recommendations, many studies have investigated the effect of diet, including reduction in SFA intake, on Lp(a) concentration. Findings from reports going back 30 years [22] to more recent studies [4,23,24,25,26,27,28] have increasingly supported a role of dietary modulation of Lp(a) levels. Several recent reviews and meta-analyses have summarized findings from randomized controlled clinical trials assessing the potential modulation of Lp(a) concentration by dietary SFA [4,27,29]. The seven trials reviewed in [4] with 15 comparisons of the effect of SFA replacement with complex carbohydrates (CHOs), monounsaturated fatty acids (MUFAs), or polyunsaturated fatty acids (PUFAs) found a variable effect on Lp(a) levels while LDL-C levels were consistently lowered. In two trials, Lp(a) increased with CHO replacement; one trial showed no effect, and another showed an Lp(a)-lowering effect [4]. MUFA replacement increased Lp(a) in three trials; three trials showed no effect, and one showed lowering. PUFA or PUFA + MUFA inconsistently affected Lp(a) in four trials. Seven trials of diets with differing macronutrient compositions showed similar divergence in the effect on LDL-C and Lp(a). It was concluded that the identified clinical trials show that diet modestly affects Lp(a) concentration, often in the opposing direction to LDL-C [4]. More recently, a meta-analysis attempted to quantify the inverse relationship between SFA intake and Lp(a) concentration based on findings from randomized controlled trials comparing a lower SFA diet with a higher SFA diet among adults without atherosclerotic CVD [29]. In the identified 27 randomized controlled trials with 49 diet comparisons, the mean difference in SFA between lower- and higher-SFA diets was 7.6% of energy (E%). Overall, Lp(a) concentration was found to be increased on average by 5.5% during lower-SFA diets compared to higher-SFA diets. This effect was primarily driven by studies using CHO or trans fatty acids (TFAs) as a replacement for SFA. Thus, replacement of SFA with CHO or TFA was estimated to increase Lp(a) concentration by 8.7% and 12.5%, respectively. Replacing SFA with MUFA or PUFA did not result in significant increases of the Lp(a) concentration, although the authors recognized uncertainty regarding effect estimates [29]. Overall, these findings suggest that lowering of SFA impacts Lp(a) concentrations, replacement strategies do matter, and that CHO and TFA, but not likely unsaturated fats, moderately increase Lp(a) concentrations.

Several factors may contribute to explaining the variability in findings in these randomized controlled clinical trials regarding the effect of diet interventions on Lp(a) concentrations. First, the use of different assays and methods for Lp(a) measurements, including enzyme-linked immunosorbent assays, which could differ by their antibody properties, immunoturbidimetry, radioimmunoassay, nephelometry, and the vertical auto profile (VAP) methods, may help explain some variability [4,29]. In particular, the VAP method used in a few studies reporting reductions in Lp(a) concentrations with SFA lowering [30,31] (included in the above review [4]) uses ultracentrifugation to quantify lipoprotein concentrations based on flotation rate [32]. This method measures cholesterol concentration of Lp(a) particles, which has a poor correlation with Lp(a) mass, likely due to the potential for overlapping with other lipoproteins [32] as well as the large interindividual variability seen with the cholesterol content of Lp(a) relative to its mass, ranging from 5.8% to 57.3% [33]. Second, the wide range of diet duration ranging from 14 days to >110 days may play a role [29]. Of note, responses of Lp(a) concentrations to SFA reduction compared to an average American diet (AAD) was found to be stable during the 5th through the 8th week of intervention [23,24,25]. In many trials, assessment of Lp(a) was a secondary outcome. Third, variability in macronutrient profiles and SFA sources and replacements across diet interventions should also contribute. Fourth, differences in cohort characteristics, including the wide age range (mean age: 22 years to >60 years), proportion of males (0% to 100%) or African Americans (0% to 100%), and the wide spectrum of the cohort sample size (n = 14 to n = 166) could contribute to the observed variability in these reported studies [4,29].

The opposite direction of responses in Lp(a) vs. LDL-C concentrations to SFA reduction was recently confirmed in a randomized controlled feeding trial of a large number of African Americans consuming a low-SFA diet (replacement with primarily CHO). In this study by Law et al. [25], the Lp(a) concentration was increased by 24% when dietary SFA was reduced from 16% in an AAD to 6% in a DASH-type diet consumed for 5 weeks. The same intervention decreased LDL-C concentration by 10%. Overall, the opposite direction and extent of changes in Lp(a) and LDL-C concentrations were in line with those in previous reports for Lp(a) (11−20% increase) and LDL-C (7−11% decrease) [23,24].

Knowledge gaps remain regarding Lp(a) properties such as its phenotypic characteristics impacting atherogenicity and its largely unknown metabolism. Therefore, a more detailed characterization of the impact of dietary changes on Lp(a)’s atherogenic properties, such as the content of oxidized phospholipids (OxPLs) and the overall lipid composition, is needed. Lp(a) was shown to be the preferential carrier of proinflammatory and proatherogenic OxPLs in plasma, carrying ~90% of OxPLs associated with apoB-containing lipoproteins, both on the apo(a) component and in the lipid phase [34,35,36]. Furthermore, a greater understanding of the heterogeneity in Lp(a) responsiveness to diet due to genetics, race/ethnicity, and metabolic and clinical phenotypes is required. Current evidence in these areas is limited.

Filling a critical knowledge gap in understanding the role of genetics in modulating Lp(a) response to diet, a recent study focused on apo(a) isoform sizes and phenotypic characteristics [37]. The study was based on the data of a large number of African Americans (n = 166) previously shown to have experienced a 24% increase in their Lp(a) concentration at the end of a 5-week DASH-type diet (SFA replaced primarily with CHO) vs. the AAD [25]. The Lp(a) response was similar for carriers and non-carriers of an atherogenic, small size apo(a), defined as ≤22 K4 repeats [38], with an increase over 20% in both groups [37]. As the median AAD Lp(a) level for carriers of a small apo(a) size was 100 mg/dL, far exceeding the 50 mg/dL threshold at which Lp(a) contributes significantly to risk [5], the Lp(a) increase in these individuals could be expected to result in a relatively greater impact on cardiovascular risk. Accordingly, DASH-type-diet-induced increases in Lp(a) resulted in a substantial shift in the distribution of Lp(a)-associated CVD risk categories [10]. For example, among the 36 individuals with an Lp(a) concentration of 30–50 mg/dL with an AAD, 18 individuals (50%) shifted their risk-associated category to the >50 mg/dL zone at the end of the DASH-type diet [37]. Also, many individuals who had an Lp(a) < 30 mg/dL with an AAD experienced a shift to the higher category, i.e., 30–50 mg/dL. Since risk estimation is based on concentrations of circulating Lp(a), these upward shifts in risk categories due to dietary changes represent a key area of interest and emphasize the importance of a precision nutrition approach in CVD prevention and management. This is especially important as an estimated 1.5−2 billion people worldwide have elevated Lp(a) > 50 mg/dL [12] and lowering of SFA intake is recommended at the population level.

In this context, it is of interest to note the recent findings showing a temporal variability in Lp(a) concentration, with greater variability in Black vs. White participants and in women than in men [39]. This observation may have some clinical implications with regard to repeated measurements of Lp(a) and may also be considered when assessing Lp(a) outcomes of targeted interventions in clinical trials, where the placebo groups could experience a temporal variability in their Lp(a). The role of diet changes in the observed temporal-related changes in Lp(a) concentrations remains unknown and should be explored in future studies.

Data regarding changes in OxPL in response to diet are very limited. A small, randomized crossover dietary trial among healthy individuals reported higher concentrations of Lp(a), OxPL/apoB, and OxPL-apo(a) during a low-fat/high-CHO diet compared with a high-fat/low-CHO diet, consumed for 4 weeks each. The high-fat/low-CHO diet was designed to provide 40%E from fat (13% SFA; 3.4% TFA) and 45%E from CHO, while the low-fat/high-CHO diet provided 20%E from fat (4.9% SFA and 2.4% TFA) and 65%E from CHO. Both diets provided 15%E from protein. As Lp(a) atherogenicity was suggested to be mediated in part via its OxPL content, there is a need to better understand diet effects on Lp(a)-OxPL.

## 3. Lp(a) and Consumption of Dietary Simple Sugars

A large body of evidence points to negative health effects of high sugar consumption and its association with CVD risk. However, less is known regarding the relationship between dietary sugar consumption and Lp(a) concentration. This issue is of particular interest as the findings described above show that replacement of SFA with CHO increases Lp(a) concentrations. In an observational study setting, participants in the top quartile of intake of sugar-sweetened beverages (SSBs) had a 20% higher relative CHD risk than those in the bottom quartile [40]. SSB consumption was associated with higher triglycerides and inflammatory biomarkers (e.g., CRP, IL-6) and lower HDL-C, consistent with results from other studies [41,42,43,44]. In contrast, there was a slight but significant decrease in Lp(a) with increased SSB consumption among the studied 1594 men (−2.81 (−4.90 to −0.72) mg/dL per 1 SSB per day increase) [40].

To further address this issue, a recent double-blind, parallel arm study among 32 overweight/obese adults tested the effect of consuming glucose- or fructose-sweetened beverages, providing 25%E intake, on Lp(a) concentration as well as the impact of apo(a) size polymorphism [28]. Following a 10-week intervention, the Lp(a) concentration was reduced by an average of 13% in all participants; notably there was no significant difference in the effect between glucose and fructose, by lower vs. higher baseline Lp(a) concentrations, or carrier status of a small size apo(a), considered to be more atherogenic than large apo(a) sizes. In contrast and similar to the results observed for SFA reduction, LDL-C responded discordantly, i.e., increased by 10% [28]. The findings that both sugars (fructose and glucose) reduced Lp(a) concentration to a similar degree raise interest considering their different metabolic pathways [45].

The exact mechanism underlying the SSB-induced decrease in Lp(a) concentration requires further studies. Apo(a) is synthesized in hepatocytes, and its synthetic rate primarily determines circulating concentration of Lp(a) [46,47,48,49]. Further, apo(a) was shown to have a 2-fold extended plasma residence time compared to apoB-100, suggesting apo(a) dissociation and recycling with apo(a)-B-containing particles [50]. While SSB consumption may impact these processes, more studies are needed to better understand mechanisms contributing to the decrease in Lp(a). Nevertheless, the data originating from a well-designed clinical trial, although based on a relatively small cohort of individuals consuming SSB [28], adds support to the growing evidence that metabolic pathways influenced by dietary changes may impact Lp(a) concentration.

## 4. Lp(a) and Ketogenic Diet

The ketogenic diet (KD) has become increasingly popular in recent years due to its metabolic impact, with subsequent potential benefits for a number of clinical conditions [51,52,53,54,55,56,57,58,59,60]. The KD is characterized by an extremely low CHO intake and high fat consumption, with the amount of protein intake adjusted accordingly. In a typical KD, fats, mainly long-chain triglycerides, generally account for 80–90%E, CHO for 5–10%E, and the remainder 6–15%E is accounted for by protein [51,61,62,63]. The metabolic consequence of a KD is an increased hepatic production of ketone bodies, such as β-hydroxybutyrate, acetoacetate, and acetone [64,65]. These ketone bodies are used as a source of energy when blood glucose and insulin levels are low due to the extreme CHO restriction and increased fat consumption.

The effect of a KD on the lipid profile has been extensively studied, with many raising concerns regarding cardiovascular health and mortality based on the increase in total cholesterol and LDL-C concentrations [53,66,67,68,69]. Conversely, low-fat (LF) diets have been associated with higher triglyceride and lower HDL-C concentrations [53,67]. Addressing the impact of KDs on CVD mortality as well as on overall mortality, a 2024 study in the National Health and Nutrition Examination Survey (NHANES) demonstrated that adherence to a KD exhibited potential in reducing all-cause mortality risk, while not posing an increased threat of CVD-related fatalities [70].

Despite the KD’s increasing popularity and substantial impact on lipid homeostasis, data on its effect on Lp(a) concentration remain limited. In an inpatient randomized crossover study in 20 adults without diabetes consuming a 2-week KD (10%E from CHO, 75%E from fats) and a 2-week plant-based, LF diet (10%E from fats, 75%E from CHO), Hall et al. tested a hypothesis that low-CHO diets (i.e., the KD) reduce ad libitum %E intake as compared to LF, high-CHO diets [71]. However, it was found that the LF diet led to less %E intake than the KD, leading to an inconsistent observation. In this study, the KD intervention resulted in a significant reduction in Lp(a) concentration (from a mean of 401 U/L at baseline to 286 U/L during the KD, *p* = 0.04). In contrast, Lp(a) concentration did not change following the plant-based LF diet [71]. On the other hand, adherence to a defined, plant-based diet for 4 weeks ad libitum in patients with elevated LDL-C, diabetes, hypertension, and/or obesity was associated with a decrease in Lp(a) concentration (~15%) [72]. Although not a typical KD, a low-CHO/high-SFA diet (20% CHO, 60% fat, including 21% SFA, and 20% protein) reduced Lp(a) concentration from baseline by 14.7% after a 20-week intervention among obese individuals [73]. The diet also resulted in improved insulin resistance, without adversely affecting LDL-C concentration. In an n = 1 experiment, a dynamic reproducible reduction in Lp(a) concentration by a KD diet was observed [74]. Furthermore, a recent secondary analysis of a clinical trial testing a low-CHO/high-fat diet intervention (CHO intake < 50 g/day) failed to produce results on Lp(a) due to concentrations being mostly below the detection level of the assay used [75]. These findings highlight the need for well-designed KD studies in diverse groups of individuals including those with elevated Lp(a) levels, as well as the importance of using validated methods for assessing Lp(a) concentrations [76,77,78]. The latter along with inclusion of a clear description of the method used for Lp(a) measurements would facilitate outcome comparisons across studies, figuring out potential reasons for inconsistent findings, and accuracy checking of outcomes.

## 5. Lp(a) Molecular and Metabolic Properties

**Figure 1:**
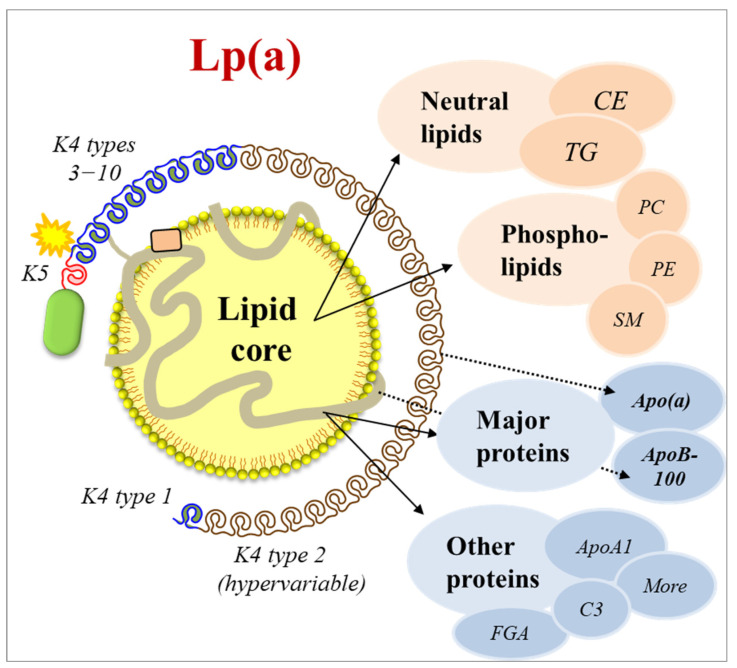
Lipoprotein(a) structure and molecular properties. Lp(a) contains a lipid core and one molecule of each of the two major apolipoproteins, apoB-100 and apo(a). Apo(a) is unique to Lp(a) with a repeated loop structure termed Kringle (K), where its K4 type 2 is present in a hypervariable copy number, resulting in an extensive apo(a) size variability. In general, smaller apo(a) sizes are associated with higher plasma Lp(a) concentrations. A set of other apolipoproteins involved in lipid metabolism as well as additional proteins linked to a variety of different functions and pathways have also been identified. The lipid core of Lp(a) consists of neutral lipids and phospholipids (PLs). While the main neutral lipids are cholesteryl esters (CEs) and triglycerides (TGs), PLs are mainly represented by choline-containing phospholipids (PCs), ethanolamine-containing phospholipids (PEs), and sphingomyelins (SMs).

The lipid core of Lp(a) consists of neutral lipids and phospholipids (PLs). While the main neutral lipids are cholesteryl esters (CEs) and TG, PLs are mainly represented by choline-containing phospholipids (PCs), ethanolamine-containing phospholipids (PEs), and sphingomyelins (SMs) (Figure 1).

The fatty acid (FA) composition varies among the neutral lipids as well as total and individual PLs and the MUFA and PUFA content relative to SFA content may impact susceptibility to oxidation [79]. Comparing lipid and FA compositions of Lp(a) vs. LDL, a previous study [80] found that the total SFAs as a percentage of the total FA pool were nearly identical for PL (55.7 vs. 54.7), PC (51.9 vs. 50.2), and PE (40.2 vs. 43.1); although it differed for TG (43.4 vs. 39.2), CE (11.3 vs. 16.8), and SM (73.2 vs. 81.2). While linoleic acid (18:2 (*n*-6)) was the major FA (>54 mol%) in CE of both Lp(a) and LDL, palmitic acid (16:0) was the major FA in TG, PL, PC, and SM for both Lp(a) and LDL [80]. Furthermore, notable differences between Lp(a) and LDL for specific lipid classes were observed. For examples, Lp(a)-PL compared with LDL-PL preferentially accumulated longer chain SFAs and MUFAs, while eicosapentaenoic acid (20:5 *n*-3) was present in LDL-TG but was absent in Lp(a)-TG. For SM, LDL-SM had a higher proportion of long-chain SFAs than Lp(a)-SM [80]. Although the significance of the FA composition in each of the lipid classes remains to be fully elucidated, it is notable that Lp(a) was shown to be a preferential carrier of OxPLs in the circulation [34] and these OxPLs predicted future cardiovascular risk [81].

Contributing to our understanding of the Lp(a) proteome, a total of 35 proteins have been identified in purified Lp(a) particles [82]. These proteins are associated with two major biological processes, i.e., lipid metabolism and wound healing. In addition to the two major Lp(a) core proteins (apoB-100 and apo(a)), a subset of apolipoproteins involved in lipid metabolism (apoC2, apoA4, apoA2, apoA1, apoL1, apoM, apoE, apoF, apoC4, and apoC3) as well as 23 additional proteins linked to a variety of different functions and pathways have been identified (Figure 1) [82]. The response to wounding was represented by proteins involved in coagulation (e.g., fibrinogen α and β), complement activation (e.g., C3), and inflammatory response (e.g., PLA_2_). These findings provided support for a previously suggested role of Lp(a) in wounding, which may point to mechanisms of Lp(a) pathogenicity at sites of vascular injury and atherosclerotic lesions.

Presently, there is a significant knowledge gap in our understanding of potential impact of diets on Lp(a) molecular properties such as its lipidome and proteome profiles. Whether dynamic qualitative and quantitative changes in specific lipid classes and FA or protein pattern associated with potential physiological functions (inflammation, coagulation, wound healing, etc.) of Lp(a) occur in response to dietary changes remains to be established. In this context, an intriguing question arises to what extent this may be the case also for LDL. Given the presence of uncertainty regarding the origin of the “LDL-like” fraction of Lp(a), such future comprehensive studies might contribute to shedding light on this issue as well as on any potential impact on atherogenicity.

## 6. Conclusions and Future Directions

**Figure 2:**
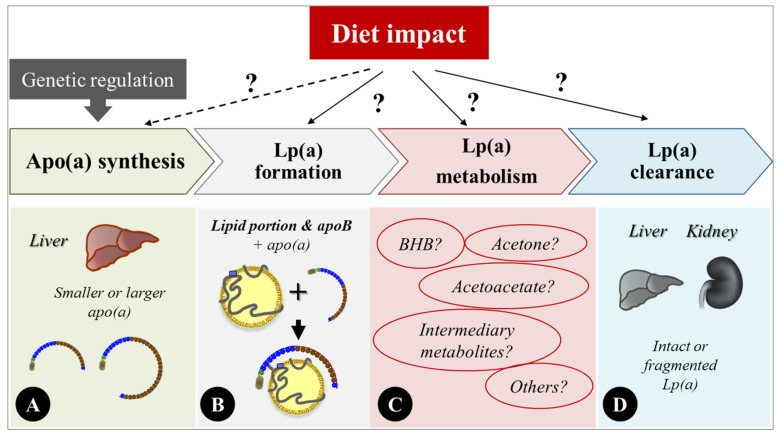
A hypothetical overview of mechanisms underlying the impact of diet interventions on Lp(a) concentrations. On one hand, the genetic regulation of Lp(a) concentrations through a copy number variation in the *LPA* gene (i.e., the apo(a) size polymorphism) is well established. Hence, apo(a) hepatic production is under strong genetic control (**panel** (**A**)). On the other hand, evidence clearly suggests a presence of metabolic regulation of Lp(a) concentration through diet changes, including reducing dietary saturated fat intake and consuming a ketogenic diet or dietary simple sugars. We suggest the possibility of a potential impact (marked with “?”) of dietary changes on: (1) Lp(a) formation, (2) Lp(a) metabolism, and/or (3) Lp(a) clearance (**panels** (**B**–**D**)). It is important to differentiate apo(a) synthesis, clearly genetically regulated (**panel** (**A**)), from Lp(a) synthesis that involves the formation of the lipid portion (**panel** (**B**)). This concept has been somewhat underappreciated and deserves more attention. It is also possible that dietary changes might impact membrane composition and therefore potentially any receptor-mediated clearance through the kidney and/or liver (**panel** (**D**)). More studies are needed to better understand the exact location(s)/phase(s) of the impact and metabolites that play roles (**panel** (**C**)) in diet-mediated effects on Lp(a) concentrations.

In this narrative review, we have provided an up-to-date summary of evidence on the role of diet in modulating Lp(a) levels. In contrast to the well-established genetic regulation of Lp(a) concentrations, any potential metabolic and environmental impact on Lp(a) concentrations or properties has garnered less attention and remains understudied. In the past 30 years since an effect of diet on Lp(a) was first reported, research interest in this area was initially less pronounced. However, findings from recent well-designed controlled feeding trials have demonstrated a clear effect of lowering dietary SFA intake on Lp(a) concentrations, where the choice of replacement macronutrients modulates the degree of change in Lp(a). Furthermore, findings in more recent studies provide support for the role of dietary simple sugars and/or KD interventions in modulating Lp(a) concentrations. In this context, the findings on complex carbohydrates vs. simple sugars are intriguing and worth pursuing to further explore whether metabolic effects might contribute. Given the increasing importance of managing Lp(a) concentrations for CVD prevention at the population level, diet modification represents an important tool. As the role of Lp(a) as a CVD risk factor, albeit an important one, must be part of a comprehensive picture, any diet guidelines need to balance effects across multiple risk factors. This complexity emphasizes the importance of research efforts to determine the detailed impact of diet variability on key Lp(a) properties and identify mechanisms underlying diet-induced effects on Lp(a) (Figure 2). Such efforts would be needed to reinforce the foundation for individualization, a necessary effort in the era of precision nutrition and precision medicine.

## Abbreviations

The following abbreviations are used in this manuscript:

Apo(a)	Apolipoprotein(a)
CHO	Carbohydrate
CVD	Cardiovascular disease
DASH	Dietary Approaches to Stop Hypertension
FA	Fatty acid
K	Kringle
KD	Ketogenic diet
Lp(a)	Lipoprotein(a)
MUFA	Monounsaturated fatty acid
PUFA	Polyunsaturated fatty acid
SFA	Saturated fatty acid
SSB	Sugar-sweetened beverage
